# Effect of intracerebroventricular (ICV) injection of antimicrobial peptide expressed in the body-2 (LEAP-2) and its interaction with cannabinoid and ghrelin systems on food intake in broiler chickens

**DOI:** 10.1016/j.psj.2025.106199

**Published:** 2025-12-06

**Authors:** Ariana Rahmania, Morteza Zendehdel, Shahin Hassanpour

**Affiliations:** a Graduate student, Faculty of Veterinary Medicine, SR.C, Islamic Azad University, Tehran, Iran; b Department of Basic Sciences, Faculty of Veterinary Medicine, University of Tehran, 14155-6453, Tehran, Iran; c Department of Veterinary Basic Sciences, SR.C., Islamic Azad University, Tehran, Iran

**Keywords:** LEAP-2, Cannabinoid, Ghrelin, Food intake, Chicken

## Abstract

Liver-expressed antimicrobial peptide 2 (LEAP-2), initially identified as an antimicrobial peptide which endocannabinoid and ghrelin action may occur via changes in Leap2 expression. This study aimed to determine effect of ICV injection of LEAP2and its interaction with cannabinoid and ghrelin systems on food intake in broiler chickens. In the present study 4 experiments were conducted, each containing 4 experimental groups. In experiment 1, chicken injected with group 1 ICV injection of saline, group 2 with LEAP2 (0.75 nmol), group 3 with LEAP2 (1.5 nmol) and group 4 with LEAP2 (3 nmol). In experiment 2, chicken received ICV injections as group 1: saline, group 2: (D-Lys-3)-GHRP-6 (0.5 nmol), group 3: LEAP2 (3 nmol) and group 4 with co injection of the (D-Lys-3)-GHRP-6 + LEAP2. In experiment 3 chiken received ICV inejctions as: group 1: saline, group 2: SR141716A (6.25 µg), group 3: LEAP2 (3 nmol) and group 4 with co injection of the SR141716A + LEAP2. In experiment 4 chiken received ICV inejctions as: group 1: saline, group 2: AM630 (1.25 µg), group 3: LEAP2 (3 nmol) and group 4 with co injection of the AM630 + LEAP2. Immediately following the infusions, the broilers were returned to their respective boxes, where they were provided with pre-weighed meal and fresh water ad libitum. The cumulative meal consumption was subsequently measured at intervals of 30, 60, and 120 min post-administration. According to the results, ICV injection of the LEAP2 (1.5 and 3 nmol) significantly decreased food intake (P<0.05). Co-injection of the (D-Lys-3)-GHRP-6 + LEAP2 significantly decreased LEAP2-induced hypophagia (P<0.05). Co-injection of the (D-Lys-3)-GHRP-6 + LEAP2 significantly increased hypophagic effect of the LEAP2 (P<0.05). Co-injection of the SR141716A + LEAP2 significantly amplified LEAP2-induced hypophagia compared to control group (P<0.05). Co-injection of the AM630 + LEAP2 significantly amplified LEAP2-induced hypophagia compared to control group (P<0.05). These results suggested that LEAP2-induced hypophagia mediates via ghrelin and CB_1_ and CB_2_ receptors in neonatal chicken.

## Introduction

The hypothalamus is crucial in regulating appetite. In the hypothalamus, incoming signals from the gastrointestinal system and brain stem are processed, along with outgoing signals for regulating food consumption. Inside the hypothalamus, numerous nuclei and neuronal pathways play roles in regulating food intake, including the Arcuate nucleus (ARC), which is a crucial hypothalamic nucleus for appetite regulation (Gan et al. 2024). The ARC houses two groups of neurons that have contrasting effects on food consumption: one group promotes food intake and expresses neuropeptide Y (NPY) along with agouti-related peptide (AgRP), while the other group inhibits feeding and co-expresses pro-opiomelanocortin (POMC) and cocaine- and amphetamine-regulated transcript (CART) (Zhang et al. 2019). Liver-expressed antimicrobial peptide 2 (LEAP-2), initially identified as an antimicrobial peptide, has recently been acknowledged as a natural inhibitor of growth hormone secretagogue receptor 1a (GHS-R1a). GHS-R1a, referred to as the ghrelin receptor, is a G protein-coupled receptor (GPCR) extensively found in the hypothalamus and pituitary gland, where it primarily regulates appetite and growth hormone (GH) release. The function of GHS-R1a is regulated by two opposing endogenous ligands: Ghrelin (activating) and LEAP-2 (inhibiting) (Lu et al. 2021).

Ghrelin is a hormone from the gut that is secreted by the stomach and influences the hypothalamus, thereby encouraging eating behavior (Mehdar, 2021). The information provided suggests that LEAP-2 and ghrelin affect the eating habits controlled by the hypothalamus. Collectively, LEAP-2 disrupts the primary role of ghrelin via interactions among the liver, stomach, and brain, and it modulates ghrelin's effects in relation to shifting environmental factors for managing food consumption, energy distribution, and homeostasis, along with the circadian rhythm (Lin and Chen, 2024). Ghrelin stimulates GHS-R1a on the NPY/AgRP neurons in the ARC to enhance appetite, and on the pituitary somatotrophs to trigger GH release. Conversely, LEAP-2 serves as both an internal competitive antagonist of ghrelin and an inverse agonist of the inherent activity of GHS-R1a. This biological characteristic of LEAP-2 effectively inhibits ghrelin's influence on food consumption and hormone release. In circulation, LEAP-2 shows an opposite trend compared to ghrelin; it rises with food consumption and obesity (positive energy balance) while it declines during fasting and weight reduction (negative energy balance) (Ma et al. 2021). A pharmacological method demonstrated that LEAP-2 and its N-terminal segment function as inverse agonists of GHSR and as competitive antagonists of inositol phosphate production and calcium mobilization triggered by ghrelin. Additionally, the N-terminal domain of LEAP-2 can suppress ghrelin-stimulated food consumption in mice, highlighting the crucial role of LEAP-2 in regulating ghrelin response in both normal and pathological scenarios (Lu et al. 2021). LEAP-2 fully inhibits GHSR activation by ghrelin and blocks the main effects of ghrelin in vivo, including promotion of food intake, GH release, and maintenance of blood glucose levels during chronic caloric restriction; however, neutralizing antibodies that block endogenous LEAP-2 function enhance the action of ghrelin in vivo (Islam et al. 2020).

The endocannabinoid system (ECS) is a signaling network made up of ECs, which are natural compounds derived from long-chain polyunsaturated fatty acids. Two cannabinoid (CB) receptors have been identified: CB1 and CB2, both of which are classified as G-protein coupled receptors (GPCRs) (Komarnytsky et al. 2021). CB1 receptors are widely found in the central nervous system. CB2 receptors are abundant in the peripheral nervous system (PNS), as well as in immune cells and tissues (Pertwee 2005), and have also been found in the brain. CB_1_ and CB_2_ receptors are believed to play a significant role in the food consumption of chickens (Alizadeh et al. 2015). There is interaction between ECS and ghrelin which ghrelin increases hypothalamic ECB levels in wild-type mice, but not in CB_1_ knockout or rimonabant-treated mice (Mattelaer et al. 2024). Moreover, CBs need the ghrelin receptor for the energy metabolism of cells induced by ghrelin (Mattelaer et al. 2024). ECS and ghrelin action may occur via changes in Leap2 expression in the liver which control of the expression of LEAP2 mediates by the by the ECBs effects on food intake and energy metabolism (Shen et al. 2021).

Despite there is report on role of the central LEAP-2 on food intake regulation in chicken, but there is no report on its interaction with cannabinoid and ghrelin systems on food intake in broiler chickens. Thus, this paper aimed to determine effect of ICV injection of antimicrobial peptide expressed in the body-2 (Leap-2) and its interaction with cannabinoid and ghrelin systems on food intake in broiler chickens.

## Material and methods

### Chicken

A total of 176 broiler chicks, one day old and of the Arian strain, were obtained from Mahan Company, a hatchery located in Tehran, Iran. For an initial two-day period, the broilers were housed together to allow for acclimatization. Subsequently, they were transferred to individual boxes. The broilers were housed in electrically heated cages to ensure a consistent temperature of 31 ± 1°C, a relative humidity between 40 % and 50 %, and a lighting regimen of 23 h of light and 1 h of darkness, following the protocol described by Olanrewaju et al. (2017). Throughout the duration of the experiment, the birds were given *ad libitum* access to a commercially prepared starter diet. This diet contained 21 % crude protein and a metabolizable energy content of 2850 kcal/kg and was sourced from the Animal Science Research Institute Co. On the fifth day post-hatch, ICV injections were administered. All procedures related to animal care and experimentation were performed in strict compliance with the guidelines specified in the Guide for the Care and Use of Laboratory Animals (National Institutes of Health, USA, publication No. 85 − 23, revised 1996) and approved by the institutional animal ethics committee.

### Drugs

In the current study, various drugs were employed, including chicken LEAP2 (amino acid sequence: MTPFWRGVSLRPVGASCRDNSECTTMLCRKNRCFLRTASE), D-Lys-3-GHRP-6 (ghrelin antagonist), SR141716A (CB_1_ cannabinoid receptor antagonist), AM630 (CB_2_ cannabinoid receptor antagonist) along with Evans Blue (Sigma, USA). Initially, all drugs were solubilized in absolute dimethyl sulfoxide (DMSO) and subsequently diluted with a 0.85 % saline solution containing Evans blue at a 1:250 ratios. This specific DMSO concentration was established in accordance with multiple prior studies (Ebrahimnejad et al. 2025). The DMSO/saline mixture with Evans blue served as the control solution in the experiment. The dosages of the drugs were determined based on previous experiments and foundational study (Lugilde et al. 2022).

### Injection procedures

In the present study 4 experiments were conducted, each containing 4 experimental groups with 11 chicks per group (total n = 176). Prior to the experiments, each chicken was weighed accurately and grouped according to body weight (BW) to ensure uniformity across treatment groups. The ICV injections were administered once per experiment using a microsyringe (Hamilton, Switzerland), without anesthesia, following the methods established by (Tachibana et al. 2022). To facilitate the injection process, an acrylic device was utilized to stabilize the chicken’s head, positioning the bill holder at a 45-degree angle and aligning the calvarium parallel to the table surface (Van Tienhoven and Juhasz 1962). An orifice was created in a plate placed over the skull to access the right lateral ventricle of the brain. The microsyringe was then inserted through this orifice, with the needle tip penetrating approximately 4 mm below the skin surface of the skull. Each injection delivered a volume of 10 μL, while the control treatment received an equivalent volume of control solution. This infusion technique has been demonstrated not to induce physiological stress in chickens (Davis et al. 1979). In experiment 1, chicken injected with group 1 ICV injection of saline, group 2 with LEAP2 (0.75 nmol), group 3 with LEAP2 (1.5 nmol) and group 4 with LEAP2 (3 nmol). In experiment 2, chicken received ICV injections as group 1: saline, group 2: (D-Lys-3)-GHRP-6 (0.5 nmol), group 3: LEAP2 (3 nmol) and group 4 with co injection of the (D-Lys-3)-GHRP-6 + LEAP2. In experiment 3 chiken received ICV inejctions as: group 1: saline, group 2: SR141716A (6.25 µg), group 3: LEAP2 (3 nmol) and group 4 with co injection of the SR141716A + LEAP2. In experiment 4 chiken received ICV inejctions as: group 1: saline, group 2: AM630 (1.25 µg), group 3: LEAP2 (3 nmol) and group 4 with co injection of the AM630 + LEAP2. Following the completion of the experiments, broilers were euthanized via decapitation in accordance with AVMA Guidelines for the Euthanasia of Animals (No: M3.6, cervical dislocation). The accuracy of each infusion was confirmed by detecting Evans blue dye in the lateral ventricle after slicing frozen brain tissue. Data analysis included only those birds that exhibited dye presence in their lateral ventricle. All experimental procedures were conducted between 08:00 and 15:30 at the animal laboratory within the Faculty of Veterinary Medicine at the University of Tehran (Table 1).

**Table 1:** Treatments procedure in experiments 1-4.

Exp. 1	ICV Injection
Treatment groups	
I	CS*
II	LEAP2 (0.75 nmol)
III	LEAP2 (1.50 nmol)
IV	LEAP2 (3 nmol)
	
**Exp. 2**	**ICV Injection**
Treatment groups	
I	CS *
II	(D-Lys-3)-GHRP-6 (0.5 nmol)
III	LEAP2 (3 nmol)
IV	(D-Lys-3)-GHRP-6 + LEAP2
	
**Exp. 3**	**ICV Injection**
Treatment groups	
I	CS *
II	SR141716A (6.25 µg)
III	LEAP2 (3 nmol)
IV	SR141716A + LEAP2
	
**Exp. 4**	**ICV Injection**
Treatment groups	
I	CS *
II	AM630 (1.25 µg)
III	LEAP2 (3 nmol)
IV	AM630 + LEAP2

CS: control solution, Liver-expressed antimicrobial peptide 2 (LEAP-2), D-Lys-3-GHRP-6 (ghrelin antagonist), SR141716A (CB1 cannabinoid receptor antagonist), AM630 (CB2 cannabinoid receptor antagonist)

### Food intake measurement

Immediately following the infusions, the broilers were returned to their respective boxes, where they were provided with pre-weighed meal and fresh water ad libitum. The cumulative meal consumption was subsequently measured at intervals of 30, 60, and 120 min post-administration. To account for variations in body weight among the subjects, meal intake was expressed as a percentage of body weight (%BW). This methodology ensures precise tracking of food intake relative to the individual weights of the chickens, thereby facilitating a more accurate assessment of their feeding behavior in response to the administered injections.

### Statistical analysis

Cumulative meal consumption, expressed as a %BW, was assessed using a one-way analysis of variance (ANOVA) and is presented as the mean ± standard error of the mean (SEM). When the ANOVA revealed significant treatment effects, pairwise comparisons among groups at each time point were conducted using the Tukey-Kramer post hoc test. The threshold for statistical significance between experimental groups was set at P < 0.05.

## Results

**Fig. 1:**
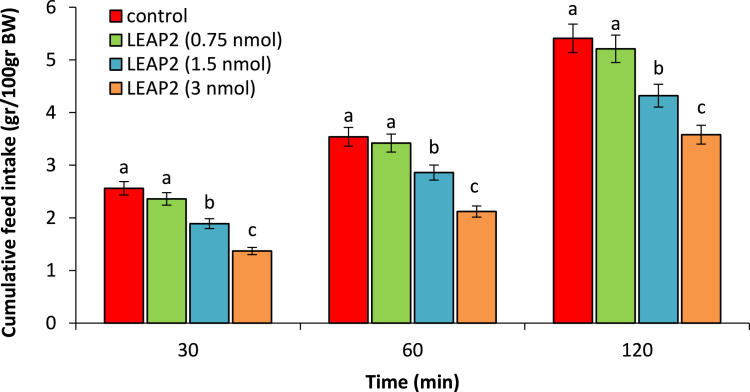
Effect of ICV injection of LEAP2 (0.75, 1.5 and 3 nmol) on cumulative food intake in neonatal chicken (n=44). Data are expressed as mean ± SEM. Different letters (a, b and c) indicate significant differences between treatments (P < 0.05).

The first experiment examined how LEAP2 influences feed intake in broiler chickens, aiming to define the effect's nature and determine an appropriate dosage. As seen in Fig. 1, ICV injection of the LEAP2 (0.75 nmol) had no significant effect on food intake compared to control group (P>0.05) while at dosages of the 1.5 and 3 nmol significantly head to hypophagia compared to control group (P<0.05).

**Fig. 2:**
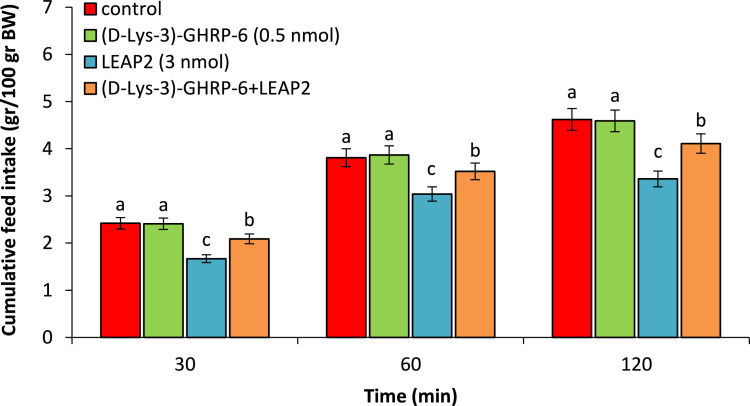
Effect of ICV injection of (D-Lys-3)-GHRP-6 (0.5 nmol), LEAP2 (3 nmol) and their combination on cumulative food intake in neonatal chicken (n=44). (D-Lys-3)-GHRP-6: ghrelin antagonist. Data are expressed as mean ± SEM. Different letters (a, b and c) indicate significant differences between treatments (P < 0.05).

As seen in Fig. 2, (D-Lys-3)-GHRP-6 (0.5 nmol) had no significant effect on food intake compared to control group (P>0.05). LEAP2 (3 nmol) significantly head to hypophagia compared to control group (P<0.05). Co-injection of the (D-Lys-3)-GHRP-6 + LEAP2 significantly decreased LEAP2-induced hypophagia compared to control group (P<0.05).

**Fig. 3:**
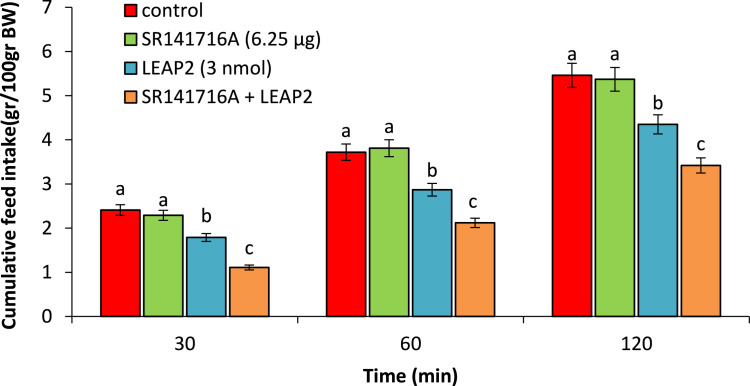
Effect of ICV injection of SR141716A (6.25 µg), LEAP2 (3 nmol) and their cssombination on cumulative food intake in neonatal chicken (n=44). SR141716A: CB_1_ cannabinoid receptor antagonist. Data are expressed as mean ± SEM. Different letters (a, b and c) indicate significant differences between treatments (P < 0.05).

As seen in Fig. 3, SR141716A (6.25 µg) had no significant effect on food intake compared to control group (P>0.05). LEAP2 (3 nmol) significantly head to hypophagia compared to control group (P<0.05). Co-injection of the SR141716A + LEAP2 significantly amplified LEAP2-induced hypophagia compared to control group (P<0.05).

**Fig. 4:**
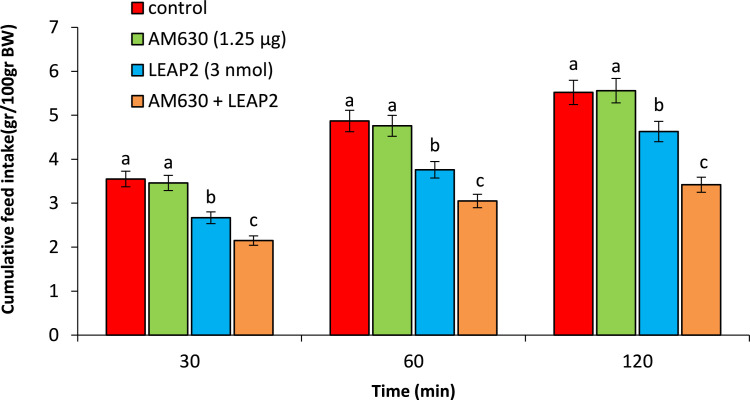
Effect of ICV injection of AM630 (1.25 µg), LEAP2 (3 nmol) and their combination on cumulative food intake in neonatal chicken (n=44). AM630: CB_2_ cannabinoid receptor antagonist. Data are expressed as mean ± SEM. Different letters (a and b) indicate significant differences between treatments (P < 0.05).

As seen in Fig. 4, AM630 (1.25 µg) had no significant effect on food intake compared to control group (P>0.05). LEAP2 (3 nmol) significantly head to hypophagia compared to control group (P<0.05). Co-injection of the AM630 + LEAP2 significantly amplified LEAP2-induced hypophagia compared to control group (P<0.05).

## Discussion

Comprehending the neuroendocrine processes that regulate appetite in broiler chickens is crucial for improving poultry science and maximizing feed efficiency (Bist et al. 2024; Guo et al. 2025). Our research offers new perspectives on the hypophagic impacts of centrally delivered LEAP2 and its relationships with ghrelin and ECB systems in broilers. Based on main findings, ICV injection of the LEAP2 head to hypophagia in chicken. LEAP2, a peptide mainly produced in liver and jejunal tissues, has been identified as an important regulator of energy balance due to its antagonistic action on the GHSR, the main receptor for ghrelin (Lu et al. 2021). In mammals, LEAP2 inhibits ghrelin-triggered appetite stimulation, elevated blood sugar, and growth hormone (GH) release (Lugilde et al. 2022). Recently, Saffar et al. (2025) reported LEAP2 injection (1.5 and 3 nmol) decreased food intake in broilers and our finding was in agreement to this report.

Co-injection of the (D-Lys-3)-GHRP-6 + LEAP2 decreased LEAP2-induced hypophagia. LEAP2 functions as an internal antagonist of the ghrelin receptor by selectively preventing ghrelin from binding to its receptor, GHSR, in a non-competitive way, thereby obstructing ghrelin-induced GH release, food consumption, and glucose mobilization (Perelló, 2025). This finding prompted the suggestion of LEAP2 as a novel possible therapeutic target for diseases related to uncontrolled ghrelin signaling, including obesity, diabetes, cachexia, and anorexia. While growing evidence suggests that ghrelin plays a key role in mammals, there is scant evidence regarding its impact on other vertebrates. Based on the reports, there are discrepancy for role of the ghrelin between avian and mammalians. Exogenous ICV delivery of ghrelin boosts food consumption in laboratory animals (Schéle et al. 2017), whereas ICV injection reduces food intake in broiler chickens (Jonaidi et al. 2012; Zendehdel et al. 2013). While the mechanisms by which broiler chickens interact with food intake are not fully understood, it has been proposed that the ARC mediates food consumption in avian species. Genetic selection has altered chicken brain neurological pathways associated with food intake. The various impacts of ghrelin on the regulation of food intake in birds and mammals are not exclusively linked to the molecular composition of ghrelin itself, and other factors may be involved, such as distinct neurological pathways. Saito et al. (2005) indicated that ICV administration of ghrelin decreases avian food consumption and elevates serum corticosterone levels. Similarly, a dose-dependent reduction in food consumption was noted following ICV injection of ghrelin in broilers (Shiimura et al. 2025). In line with this hypothesis, a notable reduction in food consumption by Japanese quail was seen following ICV injection of 1 nmol ghrelin (Shousha et al. 2005).

Based on our findings, co-injection of the AM630 + LEAP2 amplified LEAP2-induced hypophagia. Co-injection of the SR141716A + LEAP2 significantly amplified LEAP2-induced hypophagia. Certain central effects of CB1 receptors might be mediated by activating the ghrelin receptor, GHSR; additionally, the anorexigenic NAE, N-oleoyl-ethanolamine (OEA), which activates PPARα, TRPV1, and GPR119 instead of acting on cannabinoid receptors, or CB1 stimulation via endocannabinoids, is believed to either inhibit or stimulate ghrelin release from the stomach, respectively (Lillo et al. 2021). Several theories have been proposed to elucidate the mechanisms by which ECBs directly influence food consumption, although the validity of these theories is still uncertain. A proposed mechanism is that ECBs interact with CB1 receptors to influence signaling in POMC neurons, which are involved in the anorexigenic neurotransmitter in the ARC (Mazier et al. 2019). CB1 receptors demonstrate their hyperphagic function by inhibiting POMC neurons within the ARC nucleus. ICV administration of CB1 receptors elevates NPY levels (Khansari et al. 2020). Hyperphagia noted following ICV administration of CB_2_ receptors agonist in layered birds (Alizadeh et al. 2015). Emadi et al. (2011) noted an increase in food consumption after ICV injection of CB65 (a CB2 receptor agonist) in neonatal broiler chickens. Food consumption rose through both CB1 and CB2 receptors in layer-type chicks, akin to mammals but differing from broilers, where only CB2 receptors are involved in feeding (Emadi et al. 2011). In this study, Arian strain broiler chicks were utilized, and there may be a difference in the regulation of central food intake between Arian strain and ROSS-308. Perhaps the selection results in this alteration among the strains (Novoseletsky et al. 2011).

The mechanism mediated by CB1 receptors is thought to affect gastric ghrelin release and feeding behavior through the mTOR pathway (Pena-Leon et al. 2020). In mice lacking CB1, ghrelin exhibited no orexigenic effect. The pharmacological blockade of CB1 receptors suppresses ghrelin signaling on hypothalamic AMP-activated protein kinase (AMPK) activity (Farrokhi et al. 2021). Ghrelin suppresses the excitatory inputs to the CB1 receptors in the paraventricular nucleus, and this action is negated by the ICV administration of a CB1 antagonist in mice. Thus, the CBergic signaling pathway is essential for the stimulatory impact of ghrelin on AMPK activity and food consumption (Kaiya 2024). Ghrelin influences the hypothalamus through growth hormone secretagogue receptor type-1 and possibly other receptors, utilizing this pathway to promote ECBS production. The stimulating influence of ghrelin on 2-AG was inhibited by rimonabant treatment in mice. Thus, there appears to be a link between central CBergic and ghrelin neurons regarding food consumption (Kaiya 2024).

In conclusion, our results suggested that LEAP2-induced hypophagia mediates via ghrelin and CB_1_ and CB_2_ receptors in neonatal chicken. Connecting fundamental neuroendocrinology with practical poultry science, these findings provide methods to improve sustainability in livestock farming, supporting global objectives to minimize resource consumption and ecological effects. Future studies need to focus on practical applications while exploring the molecular complexities of LEAP2′s functions.

## CRediT authorship contribution statement

**Ariana Rahmania:** Data curation. **Morteza Zendehdel:** Supervision, Methodology. **Shahin Hassanpour:** Writing – review & editing, Project administration, Methodology.

## Disclosures

The authors declare for no Declaration of competing interest
